# Carvedilol inhibits EGF-mediated JB6 P+ colony formation through a mechanism independent of adrenoceptors

**DOI:** 10.1371/journal.pone.0217038

**Published:** 2019-05-20

**Authors:** Kristan H. Cleveland, Sherry Liang, Andy Chang, Kevin M. Huang, Si Chen, Lei Guo, Ying Huang, Bradley T. Andresen

**Affiliations:** 1 Department of Pharmaceutical Sciences, College of Pharmacy, Western University of Health Sciences, Pomona, California, United States of America; 2 Division of Biochemical Toxicology, National Center for Toxicological Research, US Food and Drug Administration, Jefferson, Arkansas, United States of America; University of South Alabama Mitchell Cancer Institute, UNITED STATES

## Abstract

Carvedilol is reported to prevent cancers in humans and animal models. However, a molecular mechanism has yet to be established, and the extent to which other β-blockers are chemopreventive remains relatively unknown. A comparative pharmacological approach was utilized with the expectation that a mechanism of action could be devised. JB6 Cl 41-5a (JB6 P+) murine epidermal cells were used to elucidate the chemopreventative properties of β-blockers, as JB6 P+ cells recapitulate in vivo tumor promotion and chemoprevention. The initial hypothesis was that β-blockers that are GRK/β-arrestin biased agonists, like carvedilol, are chemopreventive. Sixteen β-blockers of different classes, isoproterenol, and HEAT HCl were individually co-administered with epidermal growth factor (EGF) to JB6 P+ cells to examine the chemopreventative properties of each ligand. Cytotoxicity was examined to ensure that the anti-transformation effects of each ligand were not due to cellular growth inhibition. Many of the examined β-blockers suppressed EGF-induced JB6 P+ cell transformation in a non-cytotoxic and concentration-dependent manner. However, the IC_50_ values are high for the most potent inhibitors (243, 326, and 431 nM for carvedilol, labetalol, and alprenolol, respectively) and there is no correlation between pharmacological properties and inhibition of transformation. Therefore, the role of α1- and β2-adrenergic receptors (AR) was examined by standard competition assays and shRNA targeting β2-ARs, the only β-AR expressed in JB6 P+ cells. The results reveal that pharmacological inhibition of α1- and β2-ARs and genetic knockdown of β2-ARs did not abrogate carvedilol-mediated inhibition of EGF-induced JB6 P+ cell transformation. Furthermore, topical administration of carvedilol protected mice from UV-induced skin damage, while genetic ablation of β2-ARs increased carvedilol-mediated effects. Therefore, the prevailing hypothesis that the chemopreventive property of carvedilol is mediated through β-ARs is not supported by this data.

## Introduction

Chronic activation of the sympathetic nervous system has long been known to contribute to cardiovascular dysfunction [1]; however, it also promotes tumor progression [2–6]. In response to chronic stress, catecholamines, i.e., epinephrine and norepinephrine, are released and subsequently bind to and activate α- and β-adrenoceptors (β-ARs) eliciting the fight-or-flight response. β-AR antagonists, also called β-blockers, are widely prescribed for the treatment of hypertension, myocardial infarction, heart failure, migraine prevention, as well as other cardiovascular conditions [7]. Aside from cardiovascular effects, β-blockers are of interest due to their potential application as an adjuvant to cancer therapy. Several retrospective studies associate the use of β-blockers with slower tumor progression and prolonged survival of patients [8–10]. However, the role of β-blockers in cancer prevention is controversial as some studies suggest that the use of β-blockers reduced the incidence of cancer while others revealed no effect [11, 12]. Importantly few studies examine a single β-blocker, or compare β-blockers, which may explain discrepancies within the literature. Counter to this trend, a clinical report examining a carvedilol cohort versus a non-carvedilol cohort demonstrates a 26% reduction in the incidence of all types of cancer [9]. Additionally, carvedilol shows promise in pre-clinical models of skin cancer prevention [13, 14].

β-blockers can be classified into four pharmacological categories based on their signaling properties: neutral antagonists (the classically assumed function of an antagonist), partial and inverse agonists (determined by ligand-mediated changes in cAMP levels) [15, 16], and GRK/β-arrestin biased agonists [17]. Additionally, β-blockers show various receptor subtype selectivity against β- as well as α-ARs, and some, such as carvedilol, have various additional pharmacological actions [18]. Differences in the β-blockers pharmacological properties or receptor selectivity may explain the conflicting results from the aforementioned clinical studies linking β-blockers with cancer prevention and treatment, as most studies do not explicitly examine a single β-blocker.

Previous studies suggest that the GRK/β-arrestin biased agonists carvedilol and alprenolol prevent cancer [14, 19], but the inverse agonist atenolol and metoprolol are not as effective and ineffective, respectively [13, 19]. Additionally, our unpublished data indicated that the GRK/β-arrestin biased agonist nebivolol also prevents transformation of JB6 P+ cells. The differential effects of β-blockers and the apparent superiority of GRK/β-arrestin biased agonists as chemopreventive β-blockers initiated the current study to examine a large subset of β-blockers to determine if the chemopreventive activity is related to either or both the β-blockers assigned pharmacological category and receptor selectivity. The specific hypothesis was that GRK/β-arrestin biased agonists are unique among β-blockers in regards to chemoprevention and that biased agonism plays a role in the chemopreventive activity of carvedilol. The non-cancerous tumor promoter sensitive cell line JB6 P+ cells were utilized to test the hypothesis [20]. JB6 P+ cells express functional β2-ARs, but not β1- and β3-ARs [13], which is similar to human keratinocytes [21]. Moreover, carvedilol-mediated inhibition of epidermal growth factor (EGF) promotion of JB6 P+ cells in soft agar mimics carvedilol-mediated attenuation of UV-induced skin tumors in mice [14]. Therefore, JB6 P+ cells treated with EGF represent an excellent cellular model for screening a panel of β-blockers for their effects on the neoplastic transformation of epidermal cells.

## Methods

### Materials

Acebutolol, alprenolol, atenolol, bucindolol, carazolol, carvedilol, CGP12177, ICI 118551, isoproterenol, labetalol, metoprolol, nadolol, nebivolol, pronethalol, timolol, and 2-{[β-(4-Hydroxyphenyl)ethyl]aminomethyl}-1-tetralone hydrochloride (HEAT HCl) were purchased from Tocris (Bristol, United Kindom). Propranolol HCl and 4-hydroxycarbazole were obtained from Sigma-Aldrich (St. Louis, MO) and bupranolol was obtained from Abcam (Cambridge, UK). All compounds were dissolved in DMSO to obtain a 10 mM stock concentration, which was stored at -20°C. EGF was purchased from Peprotech (Rocky Hill, NJ) and dissolved in sterile deionized water at a 10 ug/mL stock and stored at -80°C. Primers were purchased from IDT (Coralville, IA). The lentiviral vector pLV-H1-EF1α-puro, annealing buffer, packaging vector, and polybrene were purchased from Biosettia Inc. (San Diego, CA). 4-Hydroxycarbazole (4-OHC) was purchased from Sigma Aldrich

### Cell culture

JB6 CI 41-5a (JB6 P+) cells, a mouse epidermal cell line sensitive to promotion of transformation, were purchased from American Type Culture Collection (ATCC, Manassas, VA). JB6 P+ cells were grown in Eagle’s minimum essential medium (EMEM) supplemented with 4% heat-inactivated fetal bovine serum and 1% penicillin/streptomycin and used at passage <15. All JB6 P+ cell culture materials were purchased from Genessee Scientific (San Diego, CA). HEK 293T cells were purchased from Biosettia Inc. and grown in DMEM supplemented with 200mM L-Glutamine, sodium pyruvate, MEM Non-essential amino acids, and 10% FBS obtained from Gibco/Invitrogen (Carlsbad, CA).

### Anchorage-independent growth assays in soft agar

In a 96-well tissue culture plate, 2x10^3^ JB6 P+ cells per well were mixed with 0.33% agar suspended on top of a layer of 0.5% agar. 4% Noble agar (Sigma-Aldrich, St Louis, MO) was prepared in PBS, autoclaved and stored at 4°C. 0.5% and 0.33% agar were diluted from 4% stock using EMEM supplemented with 10% FBS and 1% penicillin/streptomycin. Ten ng/mL EGF was used to promote the anchorage-independent growth of JB6 cells. The test compounds were added together with EGF at 0.01, 0.1, 1, 10 and 100 μM into the top and bottom layers of the agar. Plates were incubated at 37°C, 5% CO_2_ for 14 days. Colonies with greater than ten cells were counted manually under a microscope.

### SRB cytotoxicity assay

96-well plates were seeded with 3x10^3^ JB6 P+ cells per well and allowed to attach overnight. On the following day, cells were treated with test compounds for 72 hours and incubated at 37°C in 5% CO_2_/95% air. Cell viability was determined using Sulforhodamine B (SRB) assay from Sigma-Aldrich according to the manufacturer’s protocol. Cellular protein content was measured using a Bio-Tek μQuant plate reader with KCJunior software.

### MTS cell proliferation assay

JB6 P+ cells were seeded in 96-well plates at 500 cells/well and allowed to attach overnight. 24 hr after seeding, cells were treated with 0.1% DMSO or carvedilol and incubated for 1, 1.5, and 2 weeks at 37°C in 5% CO_2_/95% air. Cell viability was determined by the addition of MTS reagent according to the manufacturer’s protocol (Promega, Madison, WI) and absorbance was measured using a Bio-Tek μQuant plate reader with KCJunior software.

### Vector construction and production of lentiviral stocks

Single-stranded oligos directed towards Adrb2 were designed using Biosettia’s short hairpin RNA (shRNA) designer software and purchased from IDT. Oligos were annealed and ligated to the lentiviral vector pLV-H1-EF1α-puro purchased from Biosettia. Sh-Adrb2 was generated using the sequence 5’-AAAAGCCCTTCTTCATTGTCAATTTGGATCCAAATTGACAATGAAGAAGGGC-3’. The scramble shRNA (5’-AAAAGCTACACTATCGAGCAATTTTGGATCCAAAATTG CTCGATAGTGT AGC-3′), which did not contain significant homology to known genes, was used as a negative control. Ligated DNA was transformed into Stabl3 competent cells (Invitrogen). Single colonies were picked, grown in 8 ml LB broth overnight and plasmid DNA was isolated and purified using Qiagen’s MiniPrep kit (Germantown, MD). Plasmid DNA was screened for oligo insertions into the lentiviral vector using BamHI and SacI restriction enzymes (New England Biolabs, Ipswich, MA) and only DNA with appropriate band sizes were used for lentiviral stock production. The generated lentiviral vectors carrying shRNA sequences for Adrb2 or scrambled control were transfected into 293T cells as follows: 3 μg shRNA plasmid was mixed with 3 μg packaging plasmids (pMDL-G, pRSV-REV, and pVSV-G) with subsequent addition of Opti-MEM (ThermoFisher, Waltham, MA). In a separate mixture, Lipofectamine 2000 (Invitrogen) was incubated with Opti-MEM for 5 min, and the two mixtures were combined and incubated for 15 min and transfected into 293T cells for 48 hr. After 48 hr, supernatants containing the lentivirus were collected, aliquoted into 1.5 ml tubes and frozen at -80°C.

### Verification of effective shRNA-mediated β2-AR knockdown

Knockdown of the β2-AR via the Adrb2 shRNA lentivirus was examined via seeding 1x10^4^ JB6 P+ cells/well of a 6-well plate in standard growth media (see above). 24 hr after seeding, the growth media was removed from attached cells and replaced with 2 ml EMEM containing 4% heat-inactivated FBS without antibiotics and 8 μg/μl polybrene. 1.5 ml lentiviral shRNA stocks of either Adrb2 or scrambled control were added to appropriate wells and plates were centrifuged at 1,000 g at room temperature for 1 hr. Following centrifugation, media was replaced with 3 ml EMEM lacking antibiotics and grown at 37°C. After 2, 3, 7, and 10 days of growth, cells were trypsinized, collected and centrifuged at 300g for 5 min. Cells were washed with 1 ml PBS, and RNA was extracted using Qiagen’s RNeasy Mini kit. RNA was reverse transcribed into cDNA using Applied Biosystems cDNA reverse transcription kit (Invitrogen) and stored at -20°C for future use.

Assessment of β2-AR knockdown for JB6 P+ cells transduced with lentiviral stocks was analyzed using qPCR. Primers for Adrb2 and β-actin were purchased from IDT and run on a 4% agarose gel to ensure primer specificity. Adrb2 was detected using the primers: Forward: 5’-TGGTTGGGCTACGTCAACTC-3’ and Reverse: 5’-CCAGCTGACAAGTGTTTGGC-3’. β-actin was detected using Forward: 5’-TGAGCTGCGTTTTACACCCT-3’ and Reverse: 5’- GCCTTCACCGTTCCAGTTTT-3’; β-actin was used for normalization. For qPCR analysis, using a master mix, cDNA was combined with SYBR green supermix and DI water. The master mix was aliquoted in a 96-well plate and corresponding forward and reverse primers were added to each well. Readings were taken using Bio-Rad’s CFX96 Touch real-time PCR system and data were normalized to β-actin and expressed as a percent of the scrambled control.

### Examination of the role of adrenergic receptors

Antagonists identified in our assays as ineffective at blocking EGF-mediated transformation of JB6 P+ cells yet displaying no or little toxicity were used as competitive antagonists towards carvedilol-mediated prevention of EGF-induced transformation of JB6 P+ cells. The assay was conducted as described above for the anchorage-independent growth assays in soft agar except that 10 μM nadolol, 10 μM CGP12177, or 1 μM HEAT HCl were added to the top and bottom agar layers with 10 ng/mL EGF plus the aforementioned doses of carvedilol.

β2-AR shRNA lentivirus infected JB6 P+ cells were utilized in modified anchorage-independent growth assays in soft agar. The procedure was shortened to seven days because the shRNA-mediated β2-AR knockdown persisted up to ten days. Infection of the cells occurred as described in the verification of effective shRNA-mediated β2-AR knockdown, and the cells were grown for three days, then they were added to the agar as described in the anchorage-independent growth assays in soft agar section. The colonies were counted after seven days in culture; thus, matching the ten days of β2-AR knockdown.

### Animal models

The Western University of Health Sciences’ Institutional Animal Care and Use Committee (WesternU IACUC) approved all animal studies prior to initiating any experiments. The animal studies were carried out under WesternU IACUC recommendations and guidelines, which follow NIH guidelines. Mice had access to water and food ad libitum and housed on a 12-hour light/dark cycle with 35% humidity. Homozygous β1-AR and β2-AR null mice (Adrb1^tm1Bkk^ Adrb2^tm1Bkk^/J Stock Number 003810) were purchased from Jackson Laboratory (Bar Harbor, ME); the double knockout mice were characterized previously [22]. The double knockout mice were bred with C57BL/6J from Jackson Lab to generate a heterozygous F1 generation. The F1 generation was bred together to generate the F2 generation of which 1/16^th^ of the offspring were a β2-AR knockout mouse. SKH-1 mice purchased from Charles River (Wilmington, MA) were bred with the β2-AR knockout mice generating a second heterozygous F1 generation. The F1 generation was bred together to generate the F2 generation of which 1/16^th^ of the offspring were a hairless β2-AR knockout mouse. The F2 generation was backcrossed with a new batch of SKH-1 from Charles River to generate mice more efficiently. The mice used in this study are 75% SKH-1 and 25% mixed background. Following experimentation, and to cull the unused mice in a colony, mice were euthanized via exposure to 32% Isoflurane followed by cervical dislocation to ensure euthanization prior to disposal.

### Eight dose UV exposure

Wild type and β2-AR knockout hairless littermates of mixed gender were divided into four treatment groups (n = 3 each group, except the 4-OHC only has 2 mice): control, UV, UV + carvedilol, and UV+ 4-hydroxycarbazole (4-OHC). 4-OHC absorbs UV to the same extent as carvedilol but is not chemopreventive [14]. Five μM carvedilol or 4-OHC in 200 μL acetone was applied topically every other day for a total of four weeks. The first two weeks were pretreatment and the second two weeks were during the UV irradiation procedure, but drugs were applied the day after UV exposure to avoid sunscreen effects. All groups involved with UV were irradiated with 200 mJ/cm^2^ dose of UV every other day for a total of eight irradiations. Bi-folded epidermal thickness was measured using a digital caliper before drug treatment on non-radiation days. Three skin thickness measurements were taken along the left, middle, and right dorsal epidermal layer, and the mean thickness was recorded for each mouse.

### Statistical analysis

Concentration-response curves were normalized so that the EGF positive control on each 96-well plate was set to 100% and the control wells on each plate were set to 0%. Toxicity assays (SRB and MTS) were normalized so that the control on each plate represented 100% viability. Normalization allowed for comparison of data over a range of JB6 P+ cell cultures, passages, and experimental time for the shRNA studies, as well as control for variation in the EGF response, and importantly allowed for a direct comparison to all concentration-response experiments including the toxicity assays. Data are expressed as mean ± SD and were analyzed using GraphPad Prism version 7.02 (La Jolla, CA). All group sizes were determined via previous studies to provide statistically meaningful data. In the soft agar assays, key results were repeated with different JB6 P+ cells to confirm the results. Similarly, the shRNA studies were conducted with three independent concentration-response curves with different JB6 P+ cell stocks. Statistical analysis of raw data was conducted with NCSS 2007 (Kaysville, UT). For two sample comparisons a t-test was utilized, and when comparing multiple samples a One-, Two-, or Three-Factor ANOVA followed by Tukey-Kramer post hoc test was utilized depending on the data. For all tests, statistical difference was donated when p < 0.05. Specific statistical tests are provided in the figure legends.

In order to examine the relationship between the affinity for the β2-AR and the ability to prevent EGF-mediated transformation of JB6 P+ cells, Pearson’s correlation was utilized within GraphPad Prism. Specifically, the published Log K_d_ values for each antagonist and the experimentally derived LogIC_50_ values for proper sigmoidal concentration-response curves and LogIΔ_2*PSD_ (see next section for an explanation of LogIΔ_2*PSD_) for all data points were compared to assess the role of the β2-AR in the β-blockers anti-neoplastic effects. Additionally, the predicted LogP score (XLogP3), a measure of lipophilicity, was obtained from PubChem and compared to the LogIΔ_2*PSD_ via a Pearson’s correlation within GraphPad Prism.

### Generation of a method to compare sigmoidal and non-sigmoidal data sets

Problems arose in the analysis of the data. A method was needed to compare the pharmacological concentration-response inhibitory curves, which are sigmoidal on a semi-log scale, to the SRB data that did not fit to a sigmoidal curve. Additionally, not all GraphPad Prism fitted data was appropriate as the predicted bottom of the curve or IC_50_ was calculated as large negative values, which is impossible for cell counts, and thus the IC_50_ could not be used to compare to proper sigmoidal concentration-response curves. To approach these problems, we developed a novel index similar to the IC_50_ that we term inhibitory change of two times the pooled standard deviation (IΔ_2*PSD_). Two times the standard deviation of a normal distribution encompasses 95.6% of the data leaving only 2.2% of the tails of the normal distribution. 4.4%, the addition of the two tails, is less than five percent of overlap between two distributions that we accept when stating p < 0.05. The IΔ_2*PSD_ is expressed in x-axis units (log [M]) as is an IC_50_ and it represents the first concentration where it is expected to observe a statistical decrease in the measured signal.

To calculate the IΔ_2*PSD_ the pooled standard deviation (PSD) was calculated using the following formula then multiplied by 2. $\sqrt{\frac{\sum {\left(sample-sample\;mean\right)}^{2}}{total\;\#\;of\;observations-total\;\#\;of\;groups}}$ This value was then subtracted from the top of the curve; the difference between two times the PSD and the top of the cure we will call point Y. The top of the curve was mathematically calculated by GraphPad Prism for sigmoidal and sigmoidal-like curves (those curves with negative bottoms of the curve), by running a linear regression of the upper asymptote (low concentrations) for point-to-point graphs, or by taking the average of the lowest few concentrations if the linear regression slope produced a slope statistically different from zero as denoted by the 95% confidence interfal of the slope. Using the formula provided by GraphPad Prism or a straight line for the point-to-point graphs, the X-axis value for point Y was determined. The error is ascribed to this measurement via adding and subtracting the pooled standard deviation (PSD) to/from point Y generating point Y ± PSD. The two new points were then transformed to the X-axis units as was done for point Y creating an asymmetric error in sigmoidal plots. When the error was asymmetric, the larger error value was chosen to err on the conservative side. See S1 Fig for a pictoral explination of the method. The data is presented as IΔ_2*PSD_ ± error.

## Results

### Carvedilol prevents EGF-induced transformation of JB6 P+ cells

**Fig 1:**
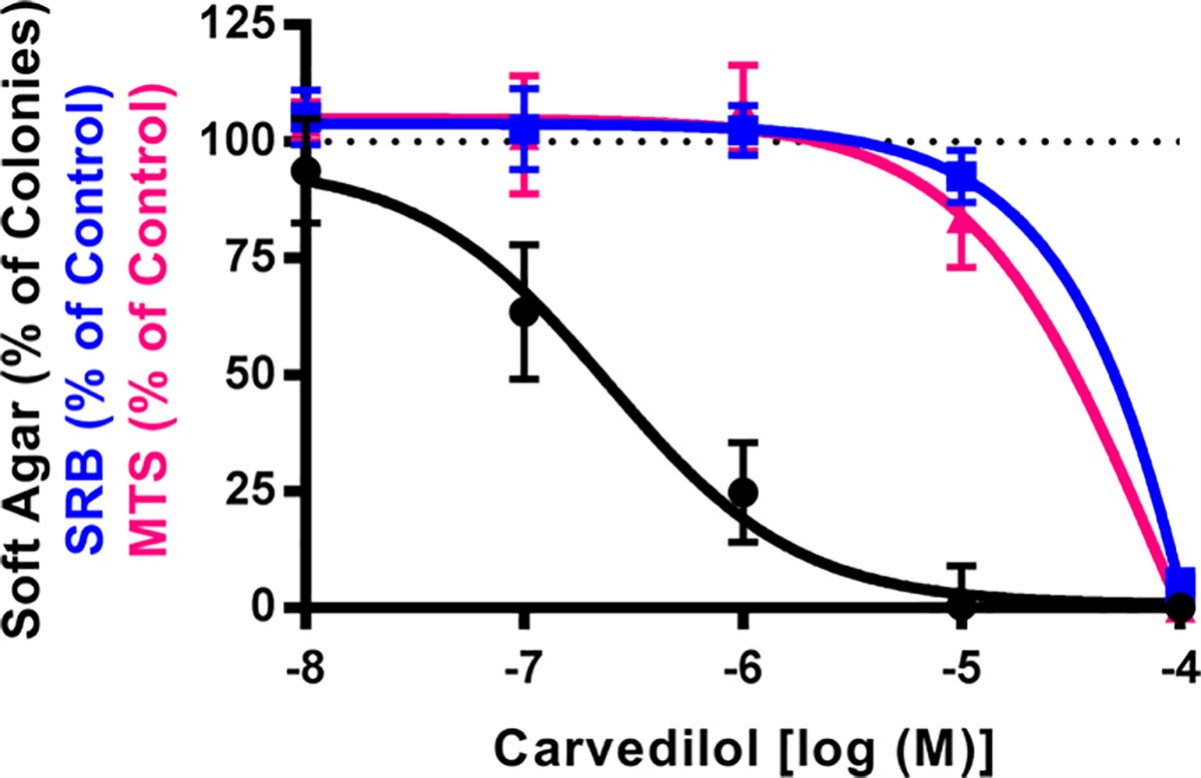
Carvedilol prevented EGF-mediated transformation of JB6 P+ cells independently of its cytotoxic effects. Examination of carvedilol in three assays: 10 μM EGF-mediated JB6 P+ cell colony formation in soft agar (black) n = 20 to 22, 72-hour SRB cell viability assay (blue) n = 6, and 2-week MTS cell viability assay (pink) n = 6. There is no statistical difference between SRB and MTS assays. Data represented as mean ± SD after normalization to control (EGF minus DMSO control for soft agar, and DMSO for SRB and MTS).

Previous data demonstrate that carvedilol completely prevents EGF-mediated transformation of JB6 P+ cells with an IC_50_ of 782 nM, whereas atenolol was less potent and displayed partial efficacy [13]. In this study, additional experiments with carvedilol (n = 20 to 22) resulted in a significantly lower IC_50_ of 243 nM (Fig 1). The IC_50_ was calculated from all the raw data generated to date from multiple stocks of JB6 P+ cells. Since an inhibitory effect could be due to toxicity, cell viability was examined using both SRB and MTS assays (Fig 1). JB6 P+ cells treated with carvedilol for two weeks, matching the transformation protocol, demonstrated cytotoxicity only at high concentrations: treatment with 100 μM carvedilol showed maximum toxicity while 10 μM is slightly (16%) toxic and concentrations lower than 10 μM did not affect cell viability. Importantly, there are no statistical differences in the viability of JB6 P+ cells treated with carvedilol for 72 hrs using the SRB assay and for 1, 1.5, and 2 weeks using the MTS assay (only the 72 hour SRB data and 2-week MTS data are shown in Fig 1). Therefore, the cytotoxicity for the remaining experiments was determined using a 72 hr SRB assay.

### Select β-AR ligands prevent EGF-induced transformation of JB6 P+ cells

**Fig 2:**
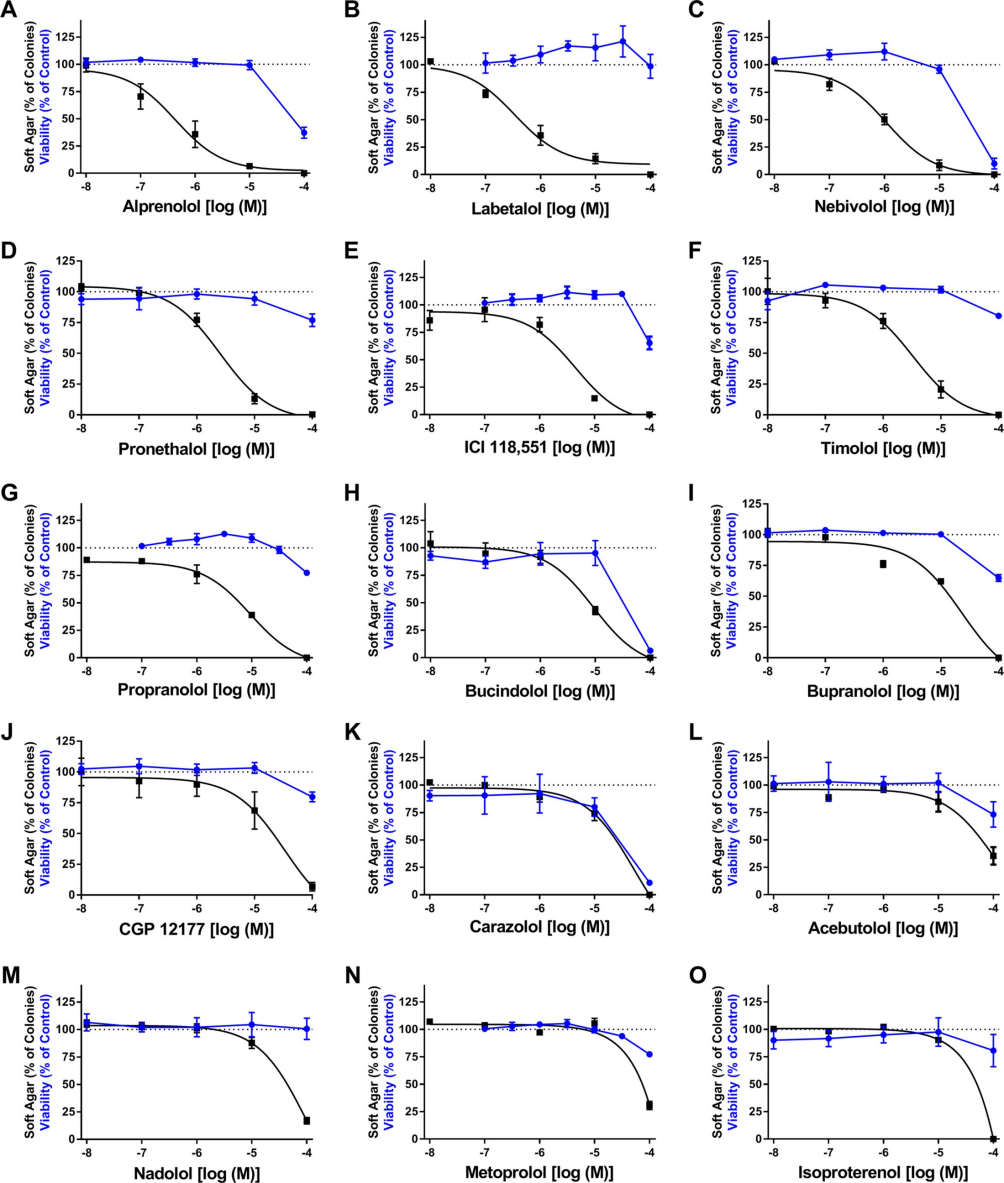
Select β-AR ligands prevent EGF-mediated neoplastic transformation of JB6 P+ cells. Exposure of JB6 P+ cells to 10 ng/mL EGF and the indicated concentration of β-AR ligands for 2-weeks in soft agar (black); n = 5 to 25. For cell viability experiments, JB6 P+ cells were exposed to the indicated concentration of β-AR ligands for 72 hrs for an SRB assay (blue); n = 6. Data represented as mean ± SD after normalization to control (EGF minus DMSO control for soft agar, and DMSO for SRB).

**Fig 3:**
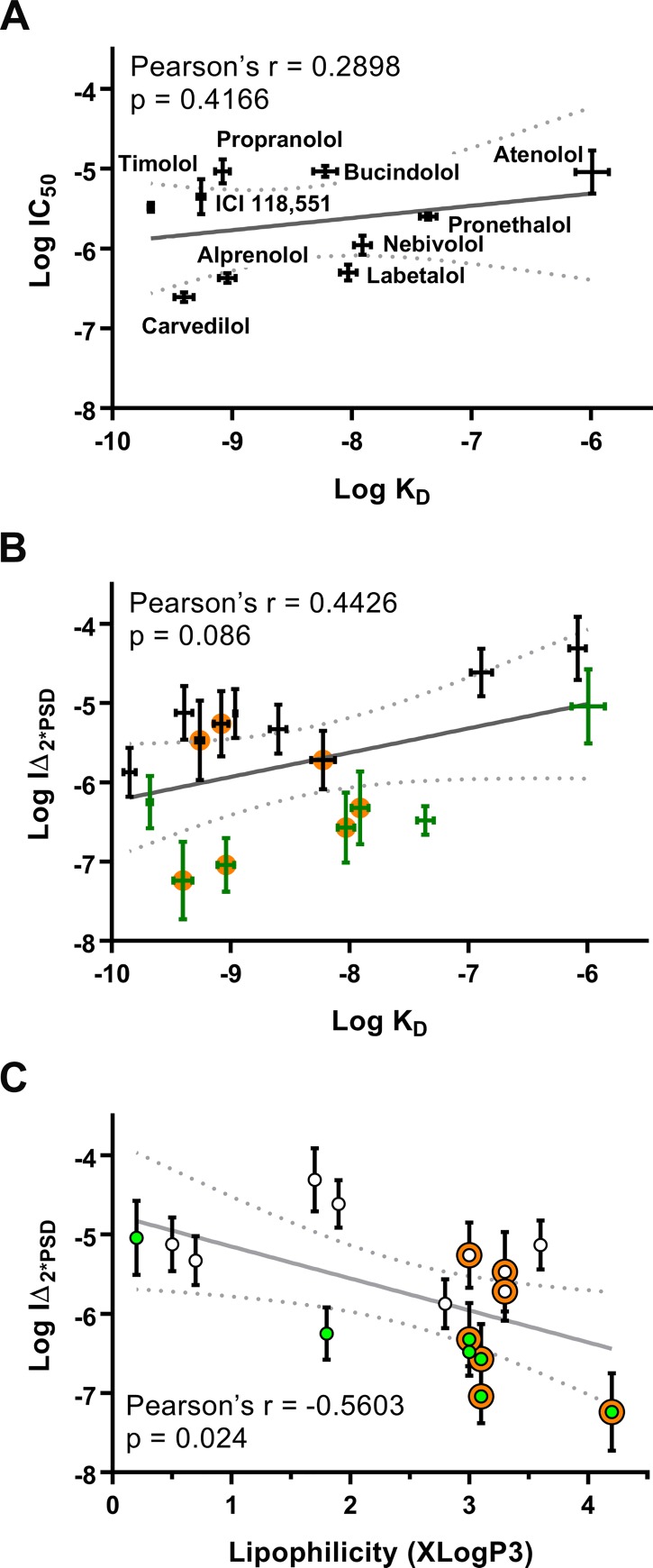
β-blocker-mediated inhibition of EGF-induced neoplastic transformation of JB6 P+ cells does not correlate with β2-AR affinity or lipophilicity. The x-axis is the reported logK_d_ values (A and B), and XLogP3 obtained from PubChem (C). The y-axis is (A) logIC_50_ and (B and C) logIΔ_2*PSD_ values for transformation. Pearson’s correlation shows that there is no correlation between log K_d_ values and either measurement, but there is a correlation between XLogP3 and logIΔ_2*PSD_. The dotted gray line represents the 95% confidence interval of the linear regression (gray line). For clarity, labels only appear in panel A. In panel B green and black symbols represent effective and non-effective β-blockers as shown in Table 1, respectively, and an orange circle represents β-blockers that are reported to have biased characteristics at the β2-AR. Panel C is similarly color-coded with open circles representing ineffective β-blockers, green circles represent effective β-blockers, and the orange halo represents β-blockers that are reported to have biased characteristics at the β2-AR.

Comparative studies were conducted using 16 β-blockers from different classes and the full β-AR agonist isoproterenol (Iso) to determine if these ligands inhibit EGF-induced JB6 P+ cell transformation (Table 1). The 16 β-blockers were chosen so that at least three ligands in each class of pharmacological properties (biased agonist, partial agonist, and inverse agonist) were examined allowing for comparative pharmacology to aid in elucidating the mechanism underlying the observed cancer preventative effects of carvedilol. Soft agar colony formation assays were conducted to evaluate the cancer preventive effects of the ligands, whereas SRB assays were used to assess their cytotoxicity. The IC_50_ and efficacy for each ligand were calculated using GraphPad Prism, and the IΔ_2*PSD_ was calculated for each experiment as described in the methods (Table 1). Since JB6 P+ cells only express β2-ARs [13], only ligand affinity (K_d_) for the β2-AR is given in Table 1. As standard sigmoidal concentration-response relationships did not appropriately model much of the SRB data and many soft agar assays, a linear point-to-point graph was used for analysis of the SRB data (Fig 2 for all ligands other than the previously published atenolol). An arbitrary cut-off of at least a10-fold difference, utilizing error propagation, between the anchorage-independent growth assay and SRB IΔ_2*PSD_ values, was denoted as effective at non-toxic concentrations. Additionally, the efficacy of the ligand, if not deemed toxic, is presented in Table 1. Carvedilol, alprenolol, and labetalol potently inhibit EGF-mediated transformation of JB6 P+ cells with IC_50_ values of 243, 431, and 326 nM, respectively, and showed little cytotoxicity within the concentration range inhibiting soft agar colony formation. Nebivolol also inhibits EGF-mediated transformation of JB6 P+ cells with an IC_50_ of 1.09 μM; however, the difference between the two IC_2*PSD_ values narrowly crosses the 10-fold cut-off. Pronethalol and timolol, which are inverse agonists, also inhibit cell transformation with an IC_50_ of 2.53 μM and 3.31 μM, respectively, with minimal toxicity. Atenolol, an inverse agonist, is the only β-blocker displaying marked partial efficacy and is the least potent of all effective β-blockers with an IC_50_ of 9.19 μM (Table 1). The remaining ligands, such as metoprolol and more so carazolol, showed inhibition of transformation closely linked to cytotoxicity of the ligand. Alternatively, the remaining ligands were non-toxic in the SRB assay but did not fit to a standard concentration-response curve and plummeted from 100% colony formation between 10 μM and 100 μM, which is well beyond therapeutic concentrations. Therefore, this data confirms that not all β-blockers are similar, but does not point to a clear mechanism underlying the cancer preventative effects as there is no correlation between receptor affinity and transformation inhibitory effect (Fig 3A and 3B). Since it was proposed that GRK/β- arrestin biased agonists are unique among β-blockers in regards to chemoprevention and that biased agonism plays a role in the chemopreventive activity, the correlation plots were color-coded with effective β-blockers colored green and biased agonists are given an orange halo around the point (Fig 3B and 3C). With this depiction of the data, it is clear that three biased ligands are ineffective in our assays and three non-biased ligands are effective. No other pharmacological property showed any prevalence for the transformation inhibitory effect. Therefore, there is also no correlation between the pharmacological properties and the ability for a β-blocker to attenuate EGF-mediated transformation of JB6 P+ cells. Additionally, activating the β-AR with isoproterenol failed to increase EGF-mediated transformation of JB6 P+ cells.

**Table 1:** Quantification and analysis of concentration-response curves of β-AR ligands.

Ligand	Pharmacological property at β2-AR	Log K_d_ at β2-AR	Soft Agar Assay		SRB Assay	
			Log IC_50_	Log IΔ_2*PSD_	Log IΔ_2*PSD_	% Efficacy/ Toxicity
Carvedilol	Biased agonist/ partial ERK agonist [43, 44]	-9.40 ± 0.08 [27]	-6.61 ± 0.06	-7.24 ± 0.49	-4.89 ± 0.09	94.65 ± 4.31
Alprenolol	Biased agonist/ activates ERK [43]	-9.04 ± 0.07 [27]	-6.37 ± 0.06	-7.04 ± 0.34	-4.95 ± 0.06	93.78 ± 2.88
Labetalol	Partial agonist for cAMP and full agonist for ERK [44, 45]	-8.03 ± 0.07 [27]	-6.30 ± 0.10	-6.57 ± 0.44	NT	90.33 ± 8.48
Nebivolol	Biased agonist/ activates ERK [46]	-7.91 ± 0.07 [47]	-5.96 ± 0.12	-6.32 ± 0.46	-4.65 ± 0.19	96.54 ± 7.24
Pronethalol	Inverse agonist for cAMP [48]	-7.36 ± 0.07 [27]	-5.60 ± 0.04	-6.48 ± 0.18	-4.86 ± 0.23	100
ICI 118,551	Inverse agonist for cAMP that recruits ±-arr [29, 49]	-9.26 ±0.03 [27]	-5.35 ± 0.22	-5.47 ± 0.50	-3.59 ± 0.50	Toxic
Timolol	Inverse agonist for cAMP [48]	-9.68 ± 0.02 [27]	-5.48 ± 0.06	-6.25 ± 0.33	-4.22 ± 0.34	100
Atenolol	Inverse agonist for cAMP [50]	-5.99 ± 0.14 [27]	-5.04 ± 0.27 [19]	-5.04 ± 0.47	NT	47.88 ± 7.81
Propranolol	Inverse agonist for cAMP that recruits ±-arr [29, 51]	-9.08 ± 0.06 [27]	-5.03 ± 0.15	-5.26 ± 0.41	-4.20 ± 0.11	Toxic
Bucindolol	Partial agonist for cAMP and full agonist for ERK [44, 52]	-8.22 ± 0.1 [52]	-5.03 ± 0.07	-5.72 ± 0.37	-4.84 ± 0.10	Toxic
Bupranolol	Antagonist, not specified	-9.85 ± 0.05 [27]	NC	-5.87 ± 0.31	-4.80 ± 0.05	Toxic
CGP 12177	Partial agonist [53]	-9.39 ± 0.07 [27]	NC	-5.12 ± 0.34	-4.46 ± 0.19	Toxic
Carazolol	Inverse agonist for cAMP [54]	-8.96 ± 0.01 [55]	NC	-5.13 ± 0.31	-5.00 ± 0.14	Toxic
Acebutolol	Partial agonist [56]	-6.08 ± 0.07 [27]	NC	-4.31 ± 0.40	-3.39 ± 0.70	Toxic
Nadolol	Inverse agonist for cAMP [44, 57]	-8.60 ± 0.07 [27]	NC	-5.33 ± 0.31	NT	ND
Metoprolol	Inverse agonist for cAMP [51]	-6.89 ± 0.09 [27]	NC	-4.61 ± 0.30	-4.52 ± 0.20	Toxic
Isoproterenol	Full equipotent agonist [44, 58]	-8.29 ± 0.02 [59]	NC	-4.61 ± 0.30	-4.77 ± 0.16	Toxic

The pharmacological properties are described as in the references; not all ligands are equally characterized. β-arr is shorthand for β-arrestin. NC indicates that the IC_50_ is not calculated because the bottom of the curve, as calculated by GraphPad Prism 7.02, is less than two times the standard deviation of the vehicle control set (-14.26%). Calculation of Log IΔ_2*PSD_ values is described in the Methods. NT indicates no toxicity at any concentration tested, and a 10-fold difference between IΔ_2*PSD_ obtained from soft agar and SRB assays is considered effective at non-toxic concentrations. If non-toxic the efficacy is reported, but if toxic it is labeled “Toxic”; ND indicates that the value could not be mathematically determined.

Since β2-ARs can signal from internal compartments [23], a correlation was run between the lipophilicity (XLogP3) and Log IΔ_2*PSD_ (Fig 3C). There is a statistical correlation (p = 0.024 and r = -0.56) between Log IΔ_2*PSD_ and lipophilicity indicating β-blockers with greater predicted lipophilicity are likely to have greater potency. However, Log IΔ_2*PSD_ does not indicate that the ligand is effective. When superimposing effectiveness onto the plot (green points), it is clear that there is no correlation between lipophilicity and effectiveness as the effective β-blockers span the lipophilicity scale (Fig 3C).

### Inhibition of adrenergic receptors does not block carvedilol from preventing EGF-induced transformation of JB6 P+ cells

**Fig 4:**
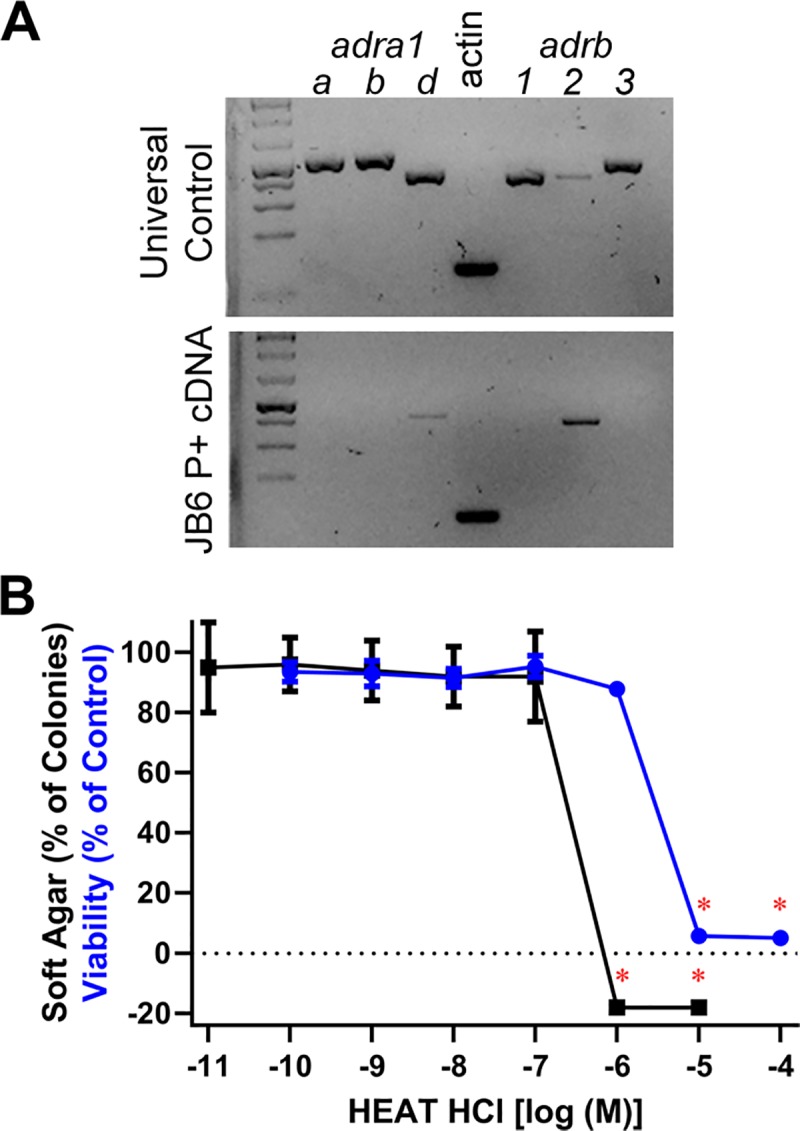
Expression of α1-ARs and role of α1-ARs in EGF-induced neoplastic transformation of JB6 P+ cells. (A) Universal mouse (positive control) and JB6 P+ cDNA was amplified with primers specific for each α1- and β-AR. Only α1D-AR and β2-AR are expressed in JB6 P+ cells. (B) 10 ng/mL EGF-mediated JB6 P+ cell colony formation in soft agar (black) n = 8 and 72-hour SRB cell viability assay (blue) n = 6. Data represented as mean ± SD after normalization to control (EGF minus DMSO control for soft agar, and DMSO for SRB and MTS). A red asterisk (*) indicate that the HEAT HCl data are statistically lower than control (p < 0.05 as per an ANOVA with Tukey-Kramer post hoc test), which is not shown for the colony formation assay, indicating toxicity.

The lack of any discernable correlation between chemopreventive activity and β2-AR affinity or efficacy as well as all experimentally derived IC_50_ values being much higher than the reported K_d_ suggests that the prevention of transformation is not due to inhibiting the β2-AR. However, carvedilol can target α1-ARs, and JB6 P+ cells express α1D-ARs as well as β2-ARs (Fig 4A). Therefore, the non-specific α1-AR antagonist HEAT HCl was examined in the soft agar and SRB assays to determine if blocking α1-ARs inhibits EGF-mediated transformation of JB6 P+ cells. As shown in Fig 4B, HEAT HCl only inhibited colony formation at 1 and 10 μM, and the data does not fit a classic sigmoidal profile. HEAT HCl-mediated inhibition of colony growth is likely due to toxicity because the SRB assay displays toxicity at 10 μM and 1 and 10 μM HEAT HCl are statistically lower than the control as determined by an ANOVA followed by a Tukey-Kramer post hoc test. Additionally, the LogIΔ_2*PSD_ is -6.84 ± 0.09 and -5.99 ± 0.03 for the soft agar and SRB assay, respectively, which is less than the 10-fold difference established to claim a pharmacological versus toxicological results.

**Fig 5:**
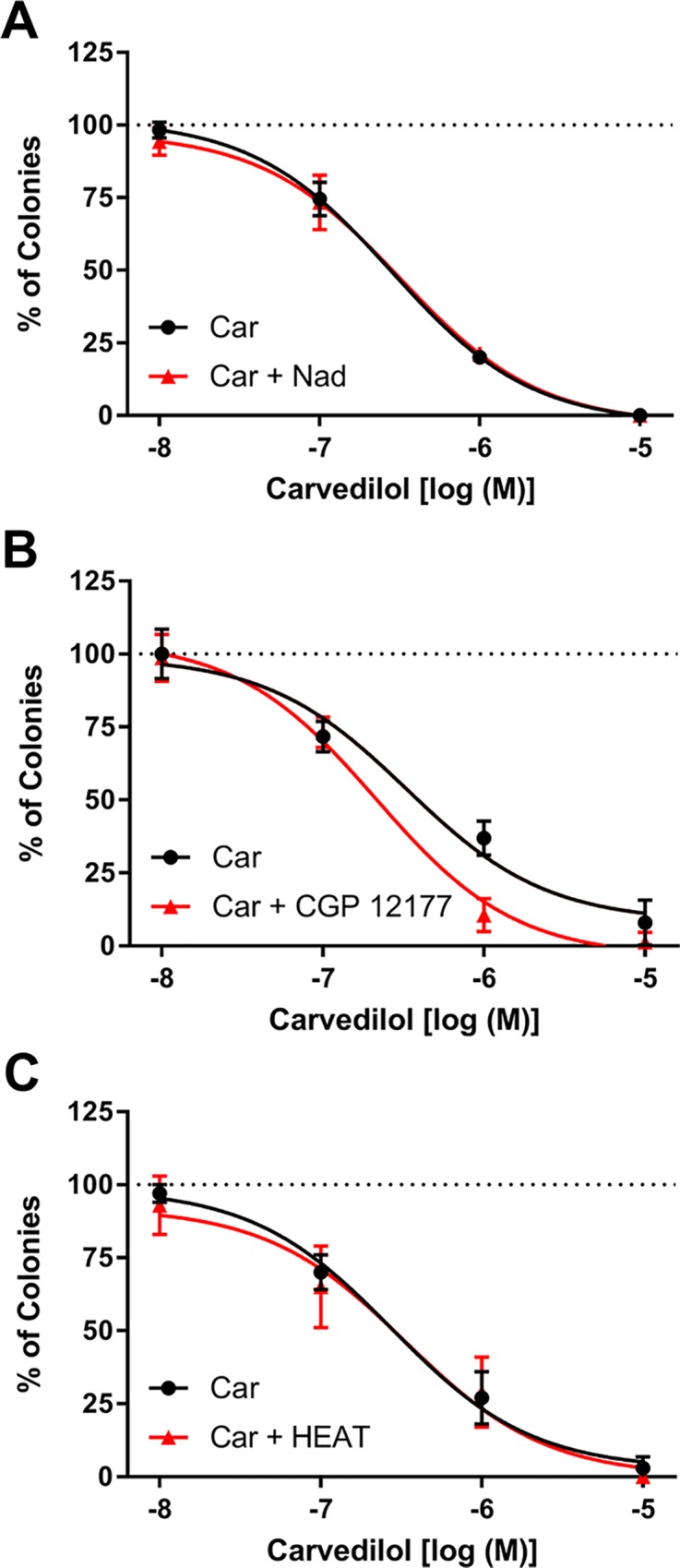
Antagonism of adrenergic receptors fails to prevent carvedilol-mediated inhibition of EGF-induced neoplastic transformation of JB6 P+ cells. JB6 P+ cells were exposed to EGF (10 ng/ml) and increasing concentrations of carvedilol (Car) in the absence and presence of (A) 10 μM nadolol, (B) 10 μM CGP 12177, or (C) 100 nM HEAT HCl. Cells were cultured for 14 days and the colonies counted under a microscope, n = 8. Data represented as mean ± SD after normalization to control (EGF alone minus DMSO control).

To confirm that adrenergic receptors are not involved in carvedilol-mediated inhibition of EGF-induced JB6 P+ cell transformation, nadolol, CGP 12177, and HEAT HCl, which do not prevent EGF-induced transformation of JB6 P+ cells (Table 1 and Figs 2 and 4), were used as competitive antagonists. Although carvedilol has nearly a 10-fold greater affinity for the β2-AR than nadolol (Table 1), 10 μM nadolol fails to attenuate any concentration of the carvedilol response (Fig 5A). The concentration-response curves are superimposable; LogIC_50_ is -6.55 ± 0.04 and -6.50 ± 0.06 for carvedilol and carvedilol plus 10 μM nadolol, respectively. Unlike nadolol, there is no significant difference between affinity towards the β2-AR of carvedilol and CGP 12177 (Table 1); however, 10 μM CGP 12177 similarly failed to attenuate the carvedilol response (Fig 5B). There is a slight shift leftwards, the opposite of inhibition, of the carvedilol concentration-response curve with CGP 12177; LogIC_50_ is -6.48 ± 0.10 and -6.67 ± 0.06 for carvedilol and carvedilol plus 10 μM CGP 12177, respectively. To examine the role of α-ARs 100 nM HEAT HCl was used as a competitive antagonist; the reported K_d_ of HEAT HCL and carvedilol towards the α1D-AR is 0.361 ± 0.08 nM and 1.2 nM, respectively [24]. As shown in Fig 5C, 100 nM HEAT HCl failed to attenuate carvedilol-mediated inhibition of EGF-induced JB6 P+ cell transformation. The concentration-response curves are nearly superimposable; LogIC_50_ is -6.56 ± 0.12 and -6.47 ± 0.13 for carvedilol and carvedilol plus 100 nM HEAT HCl, respectively. Similar studies were conducted with nebivolol and CGP 12177, which has a 30-fold greater affinity for the β2-AR compared to nebivolol (Table 1). The two treatments resulted in nearly identical concentration-response curves with the combination showing slightly greater inhibitory effects; LogIC_50_ is -6.46 ± 0.11 and -6.81 ± 0.12 for nebivolol and nebivolol plus CGP 12177, respectively (S2 Fig). Given a lack of pharmacological inhibition of carvedilol and nebivolol, the data further suggest that the β2-AR is not involved in β-blocker-mediated inhibition of EGF-induced JB6 P+ cell anchorage-independent growth.

### shRNA knockdown of β2-AR fails to alter carvedilol-mediated inhibition of JB6 P+ cell transformation

**Fig 6:**
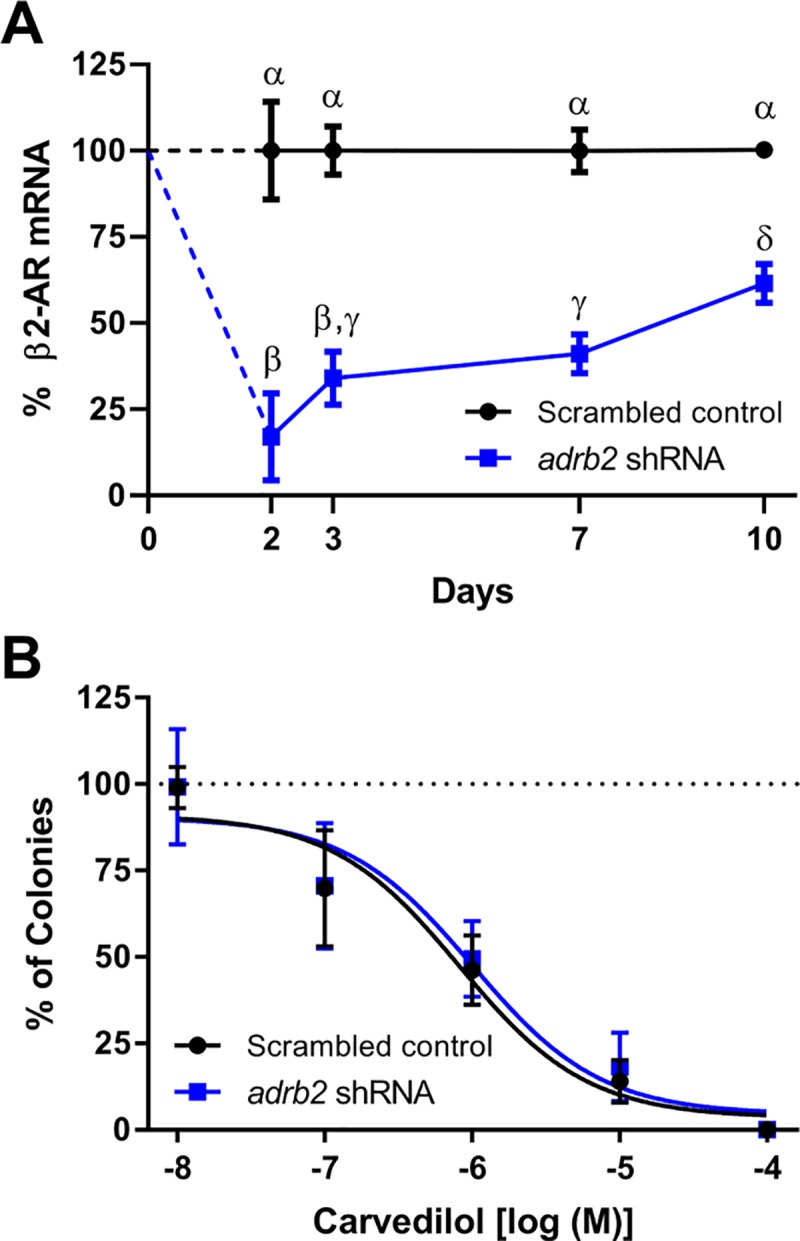
Knockdown of the β2-AR fails to prevent carvedilol-mediated inhibition of EGF-induced neoplastic transformation of JB6 P+ cells. (A) qPCR indicates that transduction of JB6 P+ cells with lentiviruses carrying a short hairpin sequence for Adrb2 decreases β2-AR expression, n = 3. Statistical analyses were determined using a 2-Factor ANOVA followed by Tukey Kramer post hoc test; different Greek letters signify statistical differences (P < 0.05). Data represented as mean ± SD. (B) After 3-days of infection, the JB6 P+ cells were exposed to 10 ng/ml EGF for seven days and increasing concentrations of carvedilol; n = 3 independent experiments with eight internal replicates detailed in Table 2. Data represented as mean ± SD after normalization to control (EGF alone minus DMSO control).

JB6 P+ cells only express the β2-AR (Fig 4A) [13]; therefore, downregulation of the β2-AR was used to further examine the role of the β2-AR in carvedilol-mediated inhibition of EGF-induced JB6 P+ cell transformation. A time course of the knockdown was conducted, demonstrating that the shRNA was maximally effective for seven days and then began to wane, but still did not reach scrambled control levels 10-days post-transduction (Fig 6A). Therefore, the soft agar assay was modified to measure colonies within seven days. Three separate experiments were conducted with eight internal replicates. The individual concentration-response curves are provided in S3 Fig, and Table 2 provides the LogIC_50_ and standard error for each experiment. Although there is slight variation in the data, a paired t-test between the LogIC_50_ of scrambled control shRNA and β2-AR-targeted shRNA demonstrates that there is no statistical difference between the two groups (p = 0.466). To graphically display the results the three experiments were combined via treating the mean of each concentration within an experiment as a single point (Fig 6B); thus, creating a data set with n = 3. The LogIC_50_ for scrambled control shRNA and β2-AR-targeted shRNA derived from Fig 6B is -6.09 ± 0.17 and -6.00 ± 0.22, respectively, demonstrating that the shRNA treatment did not affect carvedilol-mediated chemoprotection.

**Table 2:** IC_50_ values for each independent shRNA experiment.

Experiment	Scrambled shRNA			*ardb2* shRNA		
	Mean logIC_50_	SE ±logIC_50_	Points /reps	Mean logIC_50_	SE ±logIC_50_	Points /reps
1	-6.498	0.111	4/8	-6.144	0.104	4/8
2	-6.181	0.098	4/8	-5.965	0.102	4/8
3	-5.758	0.143	4/8	-5.917	0.138	4/8
Mean	-6.146	0.117		-6.009	0.114	

Three independent concentration-response experiments were conducted with scrambled and *ardb2* targeted shRNA. GraphPad Prism 7.02 was used to determine the logIC_50_ and related standard error (SE). Points/reps indicate the number of concentrations used to make the curve (points on the graph) and the number of samples in each point (reps).

### Effects of carvedilol on UV-induced skin thickening in hairless Adrb2 knockout mice

**Fig 7:**
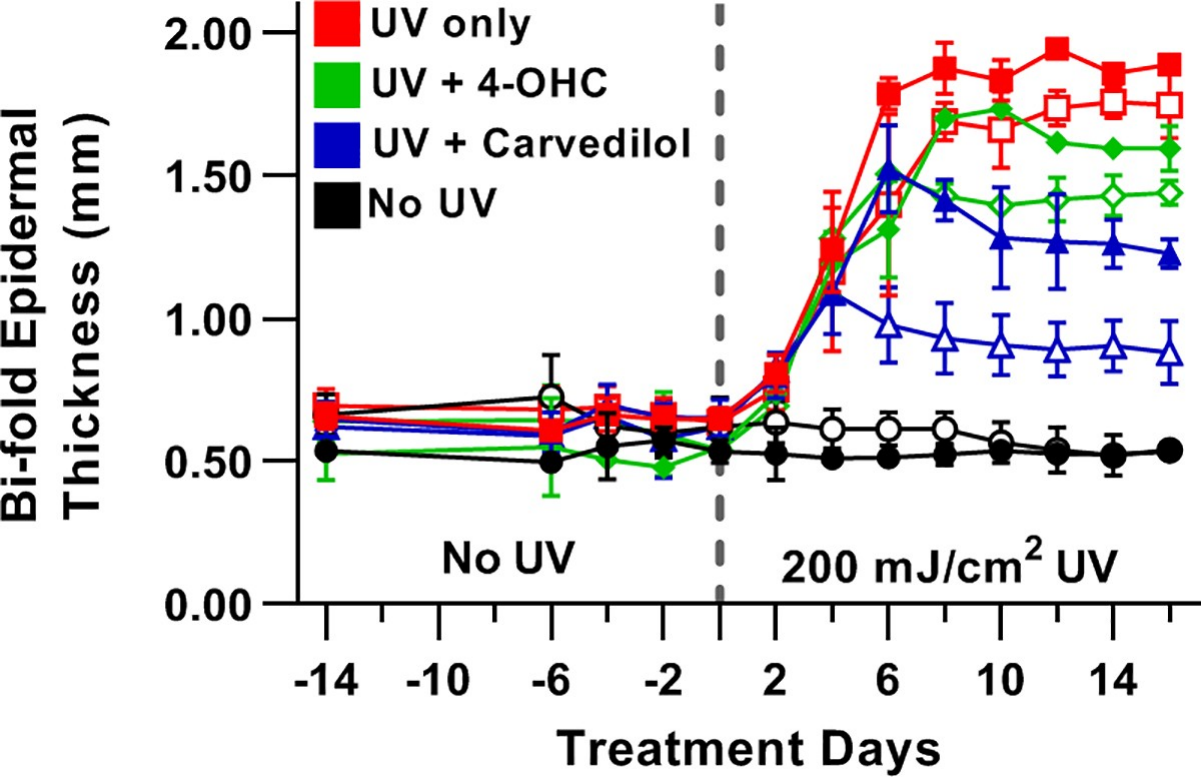
Short term UV-induced epidermal thickening in wild-type and β2-adrenergic receptor knockout mice. Hairless mice were exposed to 200 mJ/cm^2^ UV on day 1 and subsequently exposed to radiation every other day. On opposing days bi-fold epidermal thickness was measured with calipers, then the mice were treated with vehicle, 5 μM 4-OHC, or 5 μM carvedilol. Closed symbols represent wild type and open symbols knockout. Statistical analysis was conducted by a 3-Way RM-ANOVA and a Tukey-Kramer post hoc test. For clarity, statistical differences are noted in the text. Data represented as mean ± SD; n = 3, except for 4-OHC which had two mice in each group.

Since JB6 P+ cells only express functional β2-ARs [13], which is the same for the human skin keratinocytes [21], and carvedilol-mediated inhibition of EGF-induced transformation of JB6 P+ cells mimic UV-induced skin cancer in SKH-1 mice [14], SKH-1 Adrb2 knockout mice were used to study the role of the β2-AR in UV-induced skin damage. Hallmarks of UV-induced skin damage include erythema (skin reddening, a.k.a. sunburn) and skin thickening; therefore, a short duration experiment was devised to examine the effect of UV in the presence of carvedilol or 4-OHC on skin thickening in wild type and β2-AR knockout mice. 4-OHC is used in the synthesis of carvedilol [25], and 4-OHC absorbs UV radiation to the same extent as carvedilol [14]; thus 4-OHC is used as a sunscreen control. A three-way repeated measures ANOVA indicates that there is sufficient power and multiple statistically different interactions between the groups (Table 3). Specifically, 200 mJ/cm^2^ UV treatment rapidly induces skin thickening in wild type and knockout mice with statistical differentiation from controls occurring after the second dose of UV (day 4) with little difference between wild type and knockout mice over time (Fig 7). 4-OHC had little effect in wild type mice and no effect in knockout mice, as expected [14]. Conversely, carvedilol showed effects in wild type and knockout mice and unexpectedly greater effects in the knockout mice. In wild type mice, carvedilol displays a statistical difference from the UV control on day 8 onward, and also shows differences compared to 4-OHC but not as consistently. In knockout mice, carvedilol displays a statistical difference from the UV controls (both genotypes), 4-OHC treatment in the knockout mice, as well as wild type mice treated with carvedilol on day 6 onward. On day 8 onward, carvedilol treatment in knockout mice displays a statistical difference from all groups. Furthermore, the knockout mice treated with carvedilol display a unique time profile, only days 4, 6, and 8 are statistically different from day 0, and day 16 is not statistically different from any time point. Therefore, the in vivo results corroborate that carvedilol is not acting through β2-ARs.

**Table 3:** UV-induced skin thickening in hairless Adrb2 knockout mice three-way repeated measures ANOVA results.

Factor	P-value	Power
A: Genotype	0.002461	0.927886
B: Treatment	0.000000	1.000000
AB Interaction	0.000257	0.997574
D: Days	0.000000	1.000000
AD Interaction	0.000000	1.000000
BD Interaction	0.000000	1.000000
ABD Interaction	0.000111	0.999898

## Discussion

Epidemiological studies provide evidence linking the use of β-blockers with reduced risk of cancer. In particular, carvedilol has received increasing attention as an anticancer agent following a 2003 patent [26]. Preclinical studies indicate that carvedilol prevents skin cancer [13, 14] and clinical evidence suggests that carvedilol prevents many types of cancer, with pronounced efficacy towards upper gastrointestinal and lung cancers [9]. The mechanism(s) of carvedilol-mediated cancer prevention have yet to be fully explored, but it was assumed that the effects are due, at least in part, to blocking β-ARs.

Sixteen β-blockers from various classes, the full agonist isoproterenol, and the α1-AR antagonist HEAT HCl (Table 1 as well as Figs 2 and 4) were examined for cancer preventative activity using the standard JB6 P+ colony formation assay. It was expected that the resulting data would elucidate a mechanism as structurally similar β-blockers, β-blockers from the same class, or both, would display similar cancer preventative properties. However, the effective β-blockers spanned classes and did not share any structural moiety unique to the effective β-blockers that were absent in the non-effective β-blockers. Thus, the soft agar assay results (Table 1 and Fig 2) cannot be merely attributable to a class effect at the β2-AR. Moreover, the reported K_d_ values at the β2-AR do not align with the rank order of IC_50_ values obtained from the soft agar assays (Fig 3A and 3B). For instance, bupranolol, which has a log K_d_ of -9.86 ± 0.05 for the β2-AR [27], shows no relative efficacy in the JB6 P+ model, whereas labetalol, which has a log K_d_ of -8.03 ± 0.07 for the β2-AR [27], is one of the most potent inhibitors of cell transformation. The difference in affinity is nearly 100-fold; therefore, as the example illustrates, simply blocking surface β2-ARs on JB6P+ cells is not the mechanism underlying inhibition of EGF-mediated colony formation.

The co-treatment studies have a weakness, the compounds used to block carvedilol and nebivolol effects are much less lipophilic than carvedilol and nebivolol. Since β-ARs can signal within the cell [23], it is possible that both carvedilol and nebivolol are acting on internal receptors. If the effects are due to lipophilicity, then two outcomes would be expected. First, all compounds with an XLogP3 ≤ 3, the XLogP3 of nebivolol, would be effective; however, propranolol, bucindolol, ICI 118,551, and carazolol all have XLogP3 ≤ 3 and do not attenuate EGF-mediated transformation of JB6 P+ cells. A possible counter-argument is that only the biased agonists with XLogP3 ≤ 3 are effective in the assay, but propranolol, bucindolol, and ICI 118,551 are reported to have biased activity [28, 29]. Thus, dismissing the idea that biased agonism is the mechanism of carvedilol- and nebivolol-mediated inhibition of EGF-induced JB6 P+ cell transformation. The second expectation is that there would be a correlation between effective β-blockers in the colony formation assay and lipophilicity, but as seen in Fig 3C, the effective β-blockers range from carvedilol with an XLogP3 of 4.2 to Atenolol with an XLogP3 of 0.2 with each cluster of XLogP3 values having at least one effective member. Therefore, it is highly unlikely that the observed effects are due to interaction with internal receptors.

Aside from the aforementioned differences in K_d_ and lack of a correlation, pharmacological antagonism of adrenergic receptors (Fig 5) and shRNA knockdown of the β2-AR (Table 2 and Fig 6) resulted in no change in the chemopreventive effect of carvedilol. Nebivolol was also studied (S2 Fig) because nebivolol lacks activity at α-ARs and has a 30-fold separation of its affinity for the β2-AR compared to CGP 12177, K_d_ = -7.91 ± 0.07 and -9.39 ± 0.07, respectively. Even with this separation in β2-AR affinity, co-treatment with 10 μM CGP 12177 failed to attenuate the chemopreventive effect of nebivolol (LogIC_50_ is -6.45 ± 0.11 for Nebivolol and -6.81 ± 0.12 for the combination) further indicating that the observed β-blocker effect is not mediated through α1- and β-ARs. Carvedilol also targets α2-AR receptors with low affinity [24]. α2-ARs were not examined in this study because the soft agar assay logIC_50_ (-6.6 ± 0.1) is greater than the reported pK_i_ of carvedilol at the α2-ARs (α2A: -5.3 ± 0.1, α2B: -5.5 ± 0.1, α2C: -5.9 ± 0.1) [24]. Additionally, HEAT HCl binds to dopamine receptors, but with markedly lower affinity than α1-ARs [30]; suggesting, that dopamine receptors are not involved as HEAT HCl fails to prevent EGF-mediated transformation of JB6 P+ cells.

The shRNA studies have a weakness in that there is the potential that there are spare receptors and the knockdown was not sufficient to block the effect of carvedilol. Spare receptors are unlikely given the rather low level of β2-ARs expressed in JB6P+ cells [13]. However, to support the in vitro data, in vivo studies were conducted with hairless β2-AR knockout mice. As shown in Fig 7, carvedilol is more effective in reducing skin thickening in the knockout mice. The most logical rationale for carvedilol displaying greater effect in knockout mice than wild type mice is that the β2-AR in the wild type mice sequesters carvedilol from its chemopreventive target. Thus, deleting the β2-AR allows for greater concentrations of free drug to bind to the target involved in reducing skin thickening. Skin thickening precedes carcinogenesis in mice [31]; however, the short-term study design does not necessarily predict that lacking the β2-AR enhances carvedilol-mediated chemoprevention. Additional long-term UV exposure studies are required to examine chemoprevention directly in the β2-AR knockout mice. However, the knockout studies demonstrate that carvedilol effects are independent of the β2-AR.

### IΔ_2*PSD_ as a novel method for comparing data sets

The comparative studies were made possible by directly comparing the SRB cytotoxicity assay to pharmacological concentration-response curves. For many assays one, or both, data sets did not conform to a standard pharmacological concentration-response curve that generates IC_50_ values. Thus, a novel method to easily compare two different data sets was required. The data with HEAT HCl (Fig 4B) illustrates why IΔ_2*PSD_ is effective. In this data set the highest two concentrations of HEAT HCl result in no colony formation, which when normalized to control containing a low level of colony formation creates a negative number with no variance. Experience with the colony formation assay is sufficient to indicate that the lack of colonies is a sign of toxicity, but ‘experience’ is subjective and lacks scientific rigor. Additionally, the lack of variance makes the data statistically different from all other data points on the graph, including the SRB data. Thus, a standard ANOVA would differentiate the data suggesting a lack of toxicity, which, as stated, is incorrect. Although the 72-hour exposure to carvedilol in the SRB assay does not differ from a 2-week exposure to carvedilol in the MTS assay, this does not indicate that the SRB and 2-week MTS assay are equivalent for all tested compounds. To deal with this uncertainty, a 10-fold difference between the two IΔ_2*PSD_ values was set to declare a difference between two data sets and avoid false positives created by a cytotoxic effect. HEAT HCl is an example of a compound showing toxicity at 2-weeks in the colony formation assay that is absent in the 72-hour SRB assay. Fortunately, the IΔ_2*PSD_ calculation also flagged this data as toxic providing a mathematical determination of toxicity. Thus, the HEAT HCl experiment acts as an unintended internal validation of the IΔ_2*PSD_ method.

### Context of β-blockers and cancer prevention

It bears mentioning that the disparities within the literature regarding the use of β-blockers in prevention and treatment of cancer [8, 10–12] may be explained by the mixed results in the array of β-blockers presented in Table 1 and Fig 2. Most of the studies examining β-blocker usage and cancer occurrence or treatment do not specify the β-blockers that had been reviewed nor do most studies specifically study one β-blocker. Based on the volume of prescriptions and national origin, one can predict that many patients were taking atenolol or metoprolol. If a patient is taking a β-blocker that is not functional or has a very high IC_50_ for chemoprevention, such as metoprolol and atenolol, respectively (Table 1 and Fig 2), then it is unlikely that a study would observe any chemopreventive effects. Alternatively, if the majority, or all, of the patients were taking an effective β-blocker as per Table 1, then chemopreventive effects are expected as observed with carvedilol [9]. Additionally, chemoprevention and cancer therapy require different pharmacological actions; carvedilol is not effective at reducing the growth of injected A549, lung tumor, cells in vivo [13]. Thus, future retrospective studies of large populations focusing on a single drug can be conducted to confirm or refute the prediction that the β-blockers with IC_50_ values less than or equivalent to 1 μM (carvedilol, alprenolol, labetalol, and nebivolol) are effective chemoprevention agents. The aforementioned study with carvedilol [9] and the data presented herein suggest that future retrospective studies with alprenolol, labetalol, and nebivolol are warranted.

The importance of using JB6 P+ cells as a model of cancer prevention is the correlation to the prevention of UV-induced skin cancer [14]; therefore, identifying the mechanism of action of carvedilol in JB6 P+ cells may translate to clinically relevant chemoprevention assays. Although this study demonstrates that the β2-ARs are not involved in prevention of EGF-mediated JB6 P+ cell transformation, the study does not indicate that β-ARs do not play a role in preventing cancer. Epinephrine binding to β-ARs is a route of carcinogenesis [3, 4, 6, 32–34] and metastasis [35]; thus, carvedilol, and the other effective β-blockers may function in the clinic due to its yet to be identified adrenergic receptor-independent pathway as well as via blocking epinephrine effects at β-ARs. Carvedilol is known to have multiple functions aside from blocking β-ARs; carvedilol possesses antioxidative and antiproliferative properties, and it directly inhibits ryanodine receptors and thus alters cellular handling of Ca^2+^ [18, 36–39], and both properties are proposed mechanisms of inhibiting cancer [40–42]. Thus, the observed effects in this study and previous in vivo and clinical studies [9, 14] may be due to inhibition of multiple oncogenic mechanisms simultaneously.

In conclusion, this study provides evidence that a select group of β-blockers has chemopreventive properties, while most β-blockers lack such effect or display the effect only at high doses that are not likely achieved in the clinic. It is becoming evident that carvedilol is a potent cancer preventative agent; this study extends this pharmacological property to alprenolol, labetalol, and nebivolol. Importantly, these data explain past, and caution future, clinical studies that categorize all β-blockers into one category. β-blockers are radically different, and should not be categorized into one class when conducting epidemiological studies or chart-based reviews, especially regarding cancer research.

## Supporting information

S1 Fig Formula and demonstration of IΔ2*PSD calculation. The IΔ_2*PSD_ represents the first point on the x-axis with an expected statistical decrease from the top of the curve. The error was calculated based on using point Y ± PSD. Due to using PSD, IΔ_2*PSD_ and its associated error are dependent on the precision of the measurements.
(TIF)

S2 Fig Antagonism of β2-ARs fails to prevent nebivolol-mediated inhibition of EGF-induced neoplastic transformation of JB6 P+ cells. JB6 P+ cells were exposed to EGF (10 ng/ml) and increasing concentrations of nebivolol (Neb) in the absence and presence of 10 μM CGP 12177. Cells were cultured for 14 days and the colonies counted under a microscope, n = 8. Data represented as mean ± SD after normalization to control (EGF alone minus DMSO control).
(TIF)

S3 Fig shRNA knockdown of β2-AR fails to alter carvedilol-mediated inhibition of JB6 P+ cell transformation. JB6 P+ cells were infected with lentiviruses containing a scrambled shRNA or an ARDB2 (β2-AR) targeted shRNA for 3-days, then exposed to EGF (10 ng/ml) and increasing concentrations of carvedilol. Cells were cultured for seven days before counting the colonies. Each panel represents an independent experiment (n = 8). Data represented as mean ± SD after normalization to control (EGF alone minus DMSO control).
(TIF)
