# Supplementary figures and images for: Carvedilol inhibits EGF-mediated JB6 P+ colony formation through a mechanism independent of adrenoceptors
Source: PLoS One. 2019 May 20;14(5):e0217038. doi: 10.1371/journal.pone.0217038 (PMC6527222; doi:10.1371/journal.pone.0217038)

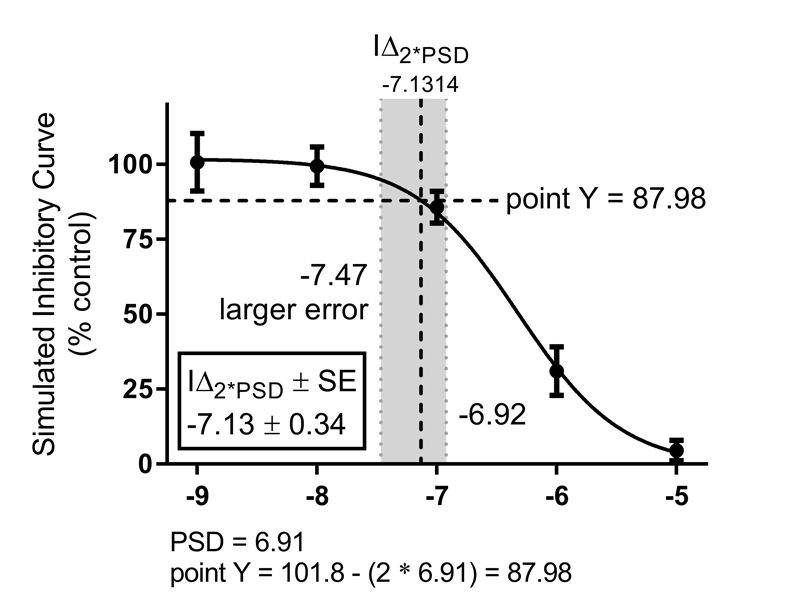

Supplement: S1 Fig — The IΔ2*PSD represents the first point on the x-axis with an expected statistical decrease from the top of the curve. The error was calculated based on using point Y ± PSD. Due to using PSD, IΔ2*PSD and its associated error are dependent on the precision of the measurements. (TIF) [file pone.0217038.s001.tif]

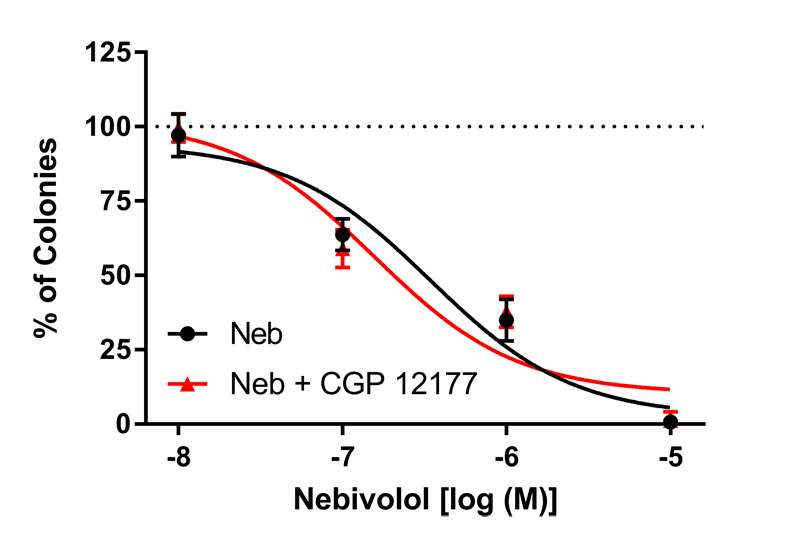

Supplement: S2 Fig — JB6 P+ cells were exposed to EGF (10 ng/ml) and increasing concentrations of nebivolol (Neb) in the absence and presence of 10 μM CGP 12177. Cells were cultured for 14 days and the colonies counted under a microscope, n = 8. Data represented as mean ± SD after normalization to control (EGF alone minus DMSO control). (TIF) [file pone.0217038.s002.tif]

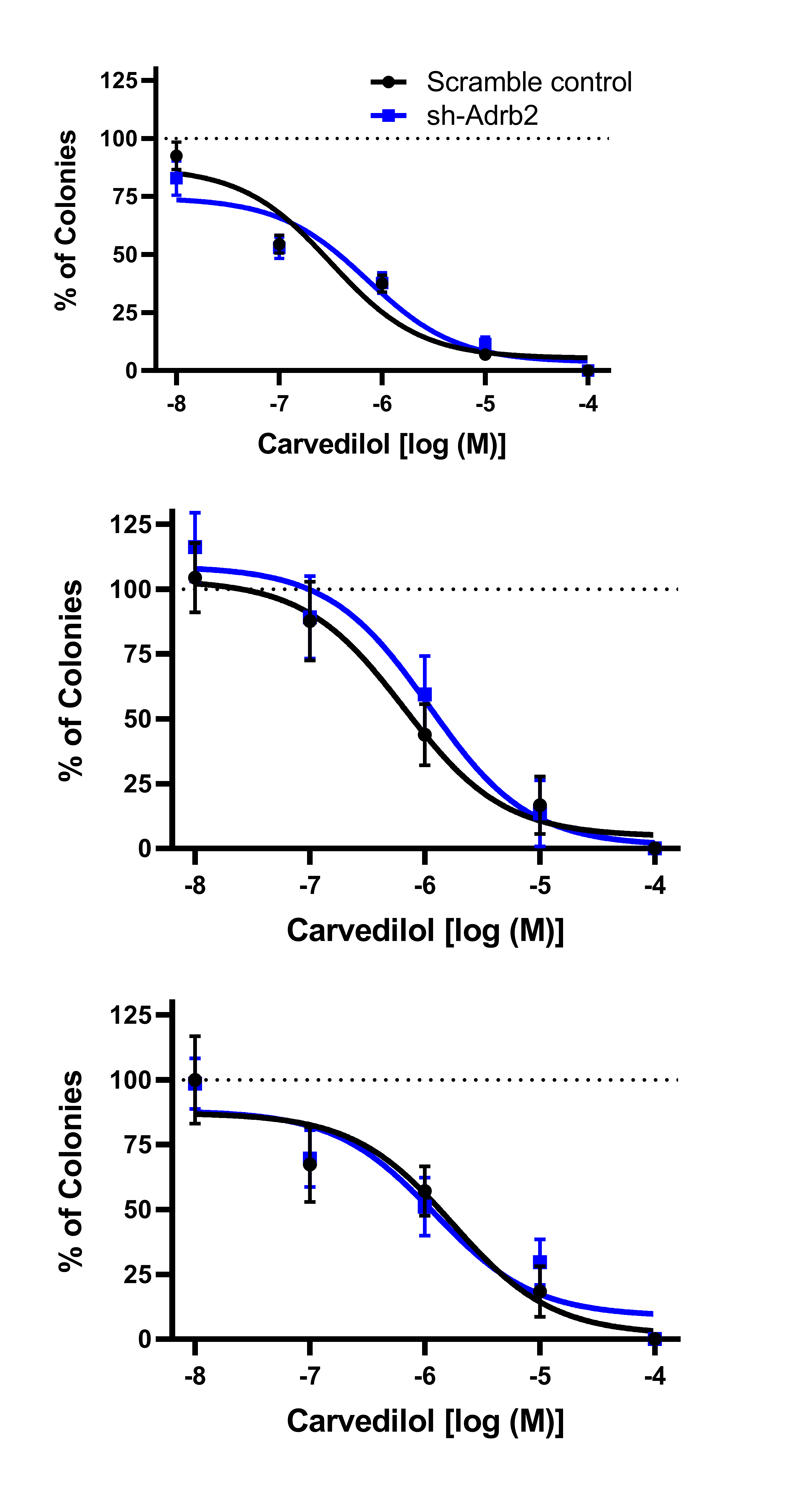

Supplement: S3 Fig — JB6 P+ cells were infected with lentiviruses containing a scrambled shRNA or an ARDB2 (β2-AR) targeted shRNA for 3-days, then exposed to EGF (10 ng/ml) and increasing concentrations of carvedilol. Cells were cultured for seven days before counting the colonies. Each panel represents an independent experiment (n = 8). Data represented as mean ± SD after normalization to control (EGF alone minus DMSO control). (TIF) [file pone.0217038.s003.tif]
